# Visual Perception and Fine Motor Skills Mediate Effects of Very Preterm Birth on Visual‐Motor Integration

**DOI:** 10.1111/apa.70474

**Published:** 2026-02-04

**Authors:** Anne‐Kathrin Dathe, Julia Jaekel, Thomas Hoehn, Ursula Felderhoff‐Mueser, Britta Maria Huening

**Affiliations:** ^1^ Department of Paediatrics I, Neonatology, Paediatric Intensive Care and Paediatric Neurology, University Hospital Essen University of Duisburg‐Essen Essen Germany; ^2^ Centre for Translational Neuro‐ and Behavioural Sciences, C‐TNBS, Faculty of Medicine University Duisburg‐Essen Essen Germany; ^3^ Department of Health and Nursing, Occupational Therapy Ernst‐Abbe‐University of Applied Sciences Jena Jena Germany; ^4^ Unit of Psychology University of Oulu Oulu Finland; ^5^ Department of Psychology University of Copenhagen Copenhagen Denmark; ^6^ Department of General Paediatrics, Neonatology and Paediatric Cardiology University Hospital Duesseldorf Düsseldorf Germany

**Keywords:** developmental profile, fine motor, preterm children, visual perception, visual‐motor skills

## Abstract

**Aim:**

The developmental profile underlying visual‐motor difficulties in very preterm children (< 32 weeks gestation) remains unclear. The aim is to test whether visual perception and fine motor skills mediate effects of very preterm birth on visual‐motor integration before school entry.

**Methods:**

60 very preterm and 60 term children were assessed at age 5–6 years with the Movement Assessment Battery for Children and the Developmental Test of Visual Perception. Direct and indirect effects of very preterm birth, visual perception and fine motor performance on visual‐motor integration were tested using mediation analysis with SPSS.

**Results:**

Mediation hypothesis was confirmed, specifically: (I) very preterm birth was associated with low visual‐motor integration, fine motor skills and visual perception (*β* = −0.46, *β* = −0.44, *β* = −0.25, *p* < 0.01, respectively). (II) Fine motor skills and visual perception were positively associated with visual motor skills (*β* = 0.62, *β* = 0.43, *p* < 0.001). (III) In the full mediation model, the direct association of very preterm birth with visual motor integration was partially mediated by fine motor skills (*β* = −0.39, *p* < 0.001) and visual perception (*β* = −0.12, *p* < 0.001).

**Conclusion:**

Effects of very preterm birth on visual‐motor integration are partially mediated by fine motor skills and visual perception. This developmental profile should be considered in screening and follow‐up assessments.

**Trial Registration:**

German clinical trial register number: DRKS00011503

## Summary

– The developmental profile underlying visual‐motor difficulties in very preterm children remains unclear.
– Very preterm birth is associated with fine motor skills, visual perception and visual‐motor integration; furthermore, visual perception and fine motor skills mediate effects of very preterm birth on visual‐motor integration.
– Combined evaluation of fine motor skills, visual perception and visual‐motor integration is helpful for identifying the developmental profile of very preterm children in follow‐up programs.

**Abbreviations**
VMI: visual‐motor integration
VP: very preterm

Preterm birth represents the largest contributor to child morbidity, with approximately 15 million (10.6%) of all newborns affected worldwide [1]. Improvements in neonatal intensive care have resulted in a reduced mortality of very preterm infants (< 32 weeks gestation, VP) in recent decades [2]. Similarly, the prevalence of severe impairments from focal lesions such as cerebral palsy has decreased [3, 4] whereas the prevalence of mild developmental delays across multiple neurodevelopmental domains remains high among VP children [5, 6]. With the decline of severe brain injury and neurological damage, milder forms of long‐term skill impairments are receiving more research attention.

A skill with high relevance to children's daily life, function and integration in social activities is visual‐motor integration (VMI). VMI is the ability to perceive and process visual input and coordinate a motor response. VMI is an umbrella term for skills such as throwing and catching a ball, spearing food onto a fork, tying shoelaces and especially guiding a pen. In the literature, VMI is usually considered to be the drawing of shapes and as a precursor skill for handwriting [7, 8]. It can predict handwriting abilities [8] and academic performance in reading and mathematics [9, 10].

Different developmental domains are involved in VMI: fine motor skills and visual perception. Fine motor skills allow picking up a pencil or pen, bringing it in position between the fingers and thumb and moving the pencil across the sheet as intended. Visual perception enables spatial orientation and detection of spatial aspects, especially to identify lines, geometric shapes and letters on a sheet. Visual perception and fine motor skills in combination empower to perform visual motor activities [11]. Preterm‐born children are at high risk of impairments in VMI [12, 13]. A review found that 40%–60% of VP children are reported to have mild or moderate deficits in fine motor skills [14]. Simultaneously, preterm children showed lower performance in visual perception and VMI with medium to large effect sizes [14, 15]. However, in most studies, each skill was examined individually and across different age ranges. Previous studies have documented associations between individual skills: Goyen and colleagues [16] have reported a high correlation between visual perception and visual motor skills as well as fine motor and VMI in VP children with a high risk profile. A further study has shown that fine motor skills mediate group differences in VMI in preterm and term‐born adolescents [17]. Previously, one group investigated all three skills at the age of 5–6 years and found that VP children were at higher risk of difficulties in visual perception, fine motor and visual‐motor skills than term‐born controls [12].

However, the specific developmental profile underlying visual motor difficulties in VP children without severe neurological impairments remains to be elucidated by investigating whether VMI is mediated by visual perception and fine motor skills. While visual perception and fine motor skills are constituent components of VMI, examining them as mediators allows us to understand whether preterm birth affects these foundational skills independently, and whether deficits in these component skills explain the observed VMI difficulties, or whether the integration process itself is additionally impaired beyond what would be expected from component skill deficits alone. We hypothesized that (I) VP birth is associated with visual perception, fine motor and VMI before school entry, (II) visual perception and fine motor skills are associated with VMI, and (III) in a full mediation model VP birth is directly and indirectly associated with VMI, mediated by visual perception and fine motor skills, after controlling for parental education and child sex.

## 1 Patients and Methods

### 1.1 Participants

This observational cohort study assessed *n* = 60 VP (*n* = 30 females) and *n* = 60 sex‐matched term born children (38 to 42 weeks gestation, *n* = 30 females) at 5–6 years [12]. VP participants were born between December 2010 and November 2012 in Level III neonatal intensive care units in a metropolitan area in Germany (University Hospital Essen and University Hospital Duesseldorf). Exclusion criteria were congenital abnormalities, severe perinatal complications (such as intraventricular haemorrhage > grade II, periventricular leukomalacia, retinopathy of prematurity > grade II) and severe cognitive impairments. Term born participants were born during the same period as the VP participants and were recruited through websites, flyers, social media and friends of participants. Full details of the recruitment strategy and exclusion criteria are provided elsewhere [12]. Dropouts and incomplete data of participants did not occur regarding primary variables.

### 1.2 Standard Protocol Approvals and Patient Consents

The study was conducted in accordance with the Helsinki Declaration and approved by local ethical committees (reference 16‐7265‐BO; 2 017 074 357). Written informed consent was obtained from parents of all participants. Children gave verbal assent.

### 1.3 Clinical Characteristics

Perinatal clinical characteristics (gestational age, birth weight, child sex) and medical complications (bronchopulmonary dysplasia, persistent ductus arteriosus, intraventricular haemorrhage, retinopathy of prematurity, Sepsis) were collected from infant medical and health records.

### 1.4 Parental Education

The highest educational qualification held by either parent was obtained via questionnaire (following the International Standard Classification of Education [18]) and binary coded into low‐medium versus high.

### 1.5 Outcome Measures

Between June 2017 and August 2018, VMI and visual perception were assessed using the German version of the Developmental Test of Visual Perception 2 (DTVP‐2) [19], and fine motor skills using the subtask Manual Dexterity of the Movement Assessment Battery for Children—second edition, German version (M‐ABC‐2) [20]. The Developmental Test of Visual Perception 2 has good reliability and high validity; the Movement Assessment Battery for Children—second edition achieves acceptable values, respectively [19, 20, 21].

### 1.6 Statistical Analysis

Descriptive characteristics were analysed with SPSS 29 (IBM Corp, NY, USA). To test the main hypotheses, mediation analyses were performed using the SPSS PROCESS macro v.4.2 according to Hayes with 5000 bootstrap resamples to test direct and indirect effects of VP birth, visual perception and fine motor performance on visual‐motor skills. Child sex and parental education were added as confounders because their influence on VMI is suspected. Therefore, we calculated both an unadjusted mediation model and a model adjusted for child sex and parental education. Sample size calculation for the two mediators' parallel mediation model was performed using Schoemann's Shinyapp power mediation. The minimum sample size was 86, with a power of 0.8 and a confidence level of 95%, assuming moderate to large effects of VP birth on VMI, fine motor skills and visual perception, based on previous evidence [12].

## 2 Results

### 2.1 Group Characteristics

Table 1 shows descriptive characteristics of *n* = 60 very preterm and *n* = 60 term born children, thus achieving the required sample size. By definition, VP children were born at a lower gestational age and birth weight than term born controls. There were no differences in child sex, parental education and age at assessment. Medical complications in the perinatal and neonatal period occurred more often in the VP group (*n* = 60) with *n* = 4 intraventricular haemorrhage grade I/II, *n* = 13 retinopathy of prematurity grade I/II, *n* = 1 mild bronchopulmonary dysplasia, *n* = 27 persistent ductus arteriosus, *n* = 12 sepsis in contrast to the term born cohort (*n* = 60) with *n* = 1 sepsis in the first weeks of life.

**TABLE 1:** Descriptive group characteristics of very preterm and term born participants.

	Very preterm (*n* = 60)	Term (*n* = 60)	*p* ^a^
Clinical characteristics			
Gestational age, weeks	28.7 [28.1–29.2]	39.5 [39.2–39.8]	**< 0.001**
Birth weight, g	1126 [1033–1219]	3414 [3287–3542]	**< 0.001**
Female, *n* (%)	30 (50)	30 (50)	1.0
Follow‐up characteristics			
Age at assessment, years	5.9 [5.8–5.9]	5.9 [5.8–6.0]	0.681
Parental education (high, *n* (%))	36 (60)	41 (68)	0.341
Visual perception, standard score	8.8 [8.0–9.6]	10.4 [9.7–11.1]	**0.004**
Fine motor skills, standard score	7.1 [6.5–7.8]	9.4 [8.8–9.8]	**< 0.001**
Visual‐motor integration, standard score	7.4 [6.8–8.0]	9.6 [9.2–10.1]	**< 0.001**

*Note:* Data are presented as mean [95% confidence interval] if not indicated otherwise. Bold values indicate *p* < 0.05.

^a^ *T*‐test and chi‐squared results for continuous and non‐continuous data, respectively.

### 2.2 Mediation Model

**FIGURE 1:**
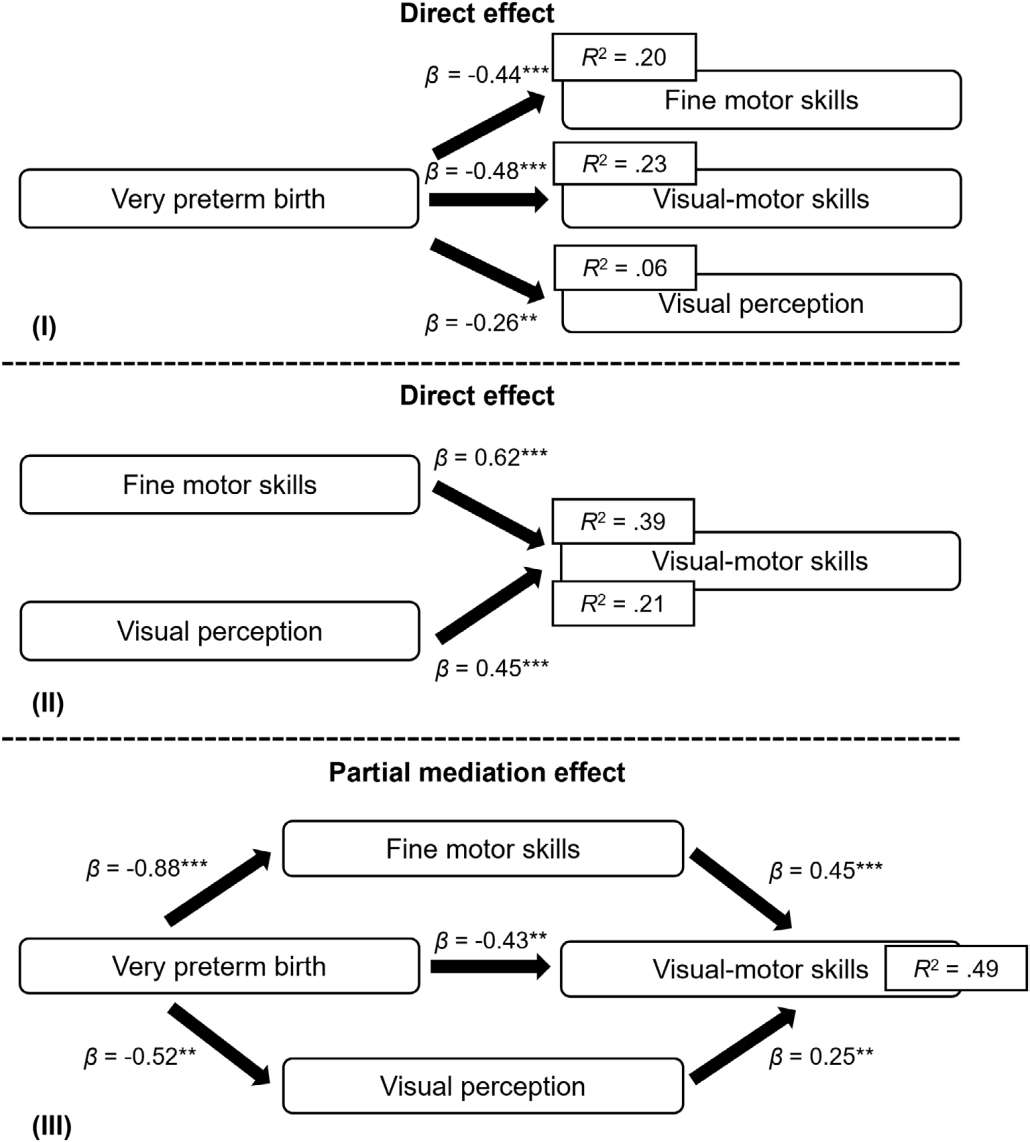
Unadjusted direct, indirect and mediation effects of very preterm birth on visual‐motor skills (*n* = 120). *p < 0.05, **p < 0.01, ***p < 0.001; *R* ^2^ = proportion of variance explained by predictor(s). This model does not control for confounders.

**FIGURE 2:**
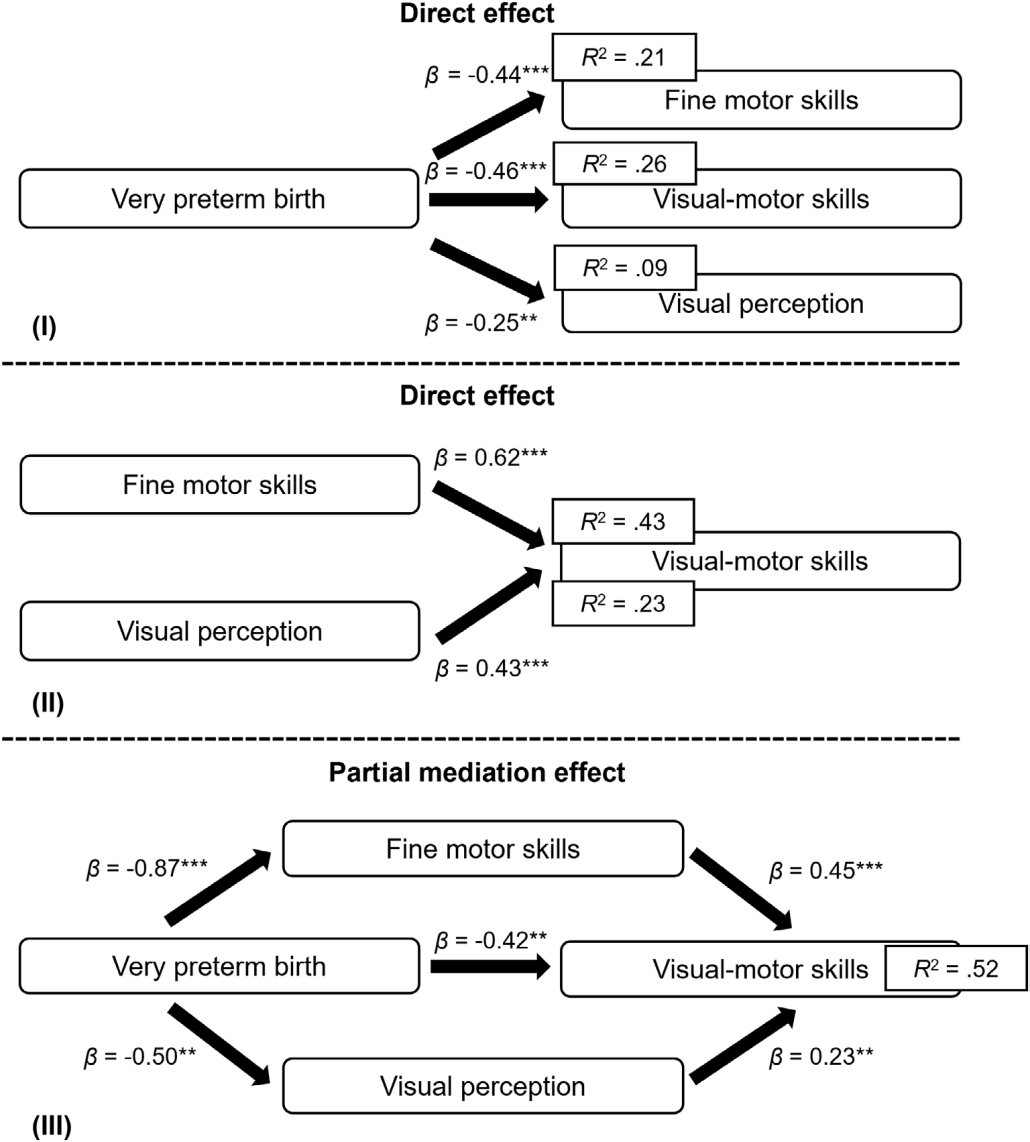
Direct, indirect and mediation effects of very preterm birth on visual‐motor skills (*n* = 120) adjusted for child sex and parental education. *p < 0.05, **p < 0.01, ***p < 0.001; *R* ^2^ = proportion of variance explained by predictor(s). This model controls for child sex and parental education as confounders.

Mediation analyses showed that all unadjusted hypothesized paths were significant (Figure 1). Models were then repeated adjusting for the confounders child sex and parental education. Both unadjusted (Figure 1) and adjusted (Figure 2) models yielded highly similar results, indicating minimal influence of child sex and parental education on the observed mediation pathways.

Figure 2 shows (I) univariate direct effects of VP birth on fine motor skills ((path *a*) *β* = −0.44, *p* < 0.001), visual motor skills ((path *b*) *β* = −0.46, *p* < 0.001) and visual perception ((path *c*) *β* = −0.25, *p* = 0.006). (II) Univariate direct effects of fine motor skills ((path *d*) *β* = 0.62, *p* < 0.001) and visual perception on visual motor skills ((path *e*) *β* = 0.43, *p* < 0.001) were confirmed. Finally, (III) the mediation model showed a direct negative effect of VP birth on visual motor skills ((path *b*) *β* = −0.42, *p* = 0.005) and standardised indirect effects of very preterm birth on VMI through fine motor skills ((path *ad*) *β* = −0.39; *p* < 0.001) and effects of very preterm birth on VMI through visual perception ((path *ce*) *β* = −0.11, *p* < 0.001). The total effect of VP birth on VMI itself and via two mediators is *β* = −0.92, *p* < 0.001. The mediation model explained 52% of the total variation in individual children's visual motor performance.

## 3 Discussion

This cohort study confirmed fine motor skills and visual perception as mediators for VMI. Results show that (I) VP birth is negatively associated with fine motor skills, VMI and visual perception. Furthermore, (II) the abilities of fine motor skills and visual perception have a positive association with VMI. (III) VP birth has a direct and indirect association with VMI, mediated by fine motor skills and visual perception, after controlling for parental education and child sex. In summary, the mediation analysis confirmed that the effect of VP birth on visual‐motor skills is partially mediated by fine motor skills and visual perception.

In this cohort, the three skills were investigated simultaneously for the first time, allowing for the examination of associations, especially mediation, which provides a more comprehensive understanding of the relationships between these skills and the impact of VP birth.

(I) Consistent with previous studies, VP children score lower in VMI, fine motor and visual perception assessments [14, 15]. Fine motor skills appear especially vulnerable, with reports of high prevalence with 40%–60% in VP children and an effect size of *d* = −0.62 for preterm birth reported in reviews [14, 22]. Visual perception deficits are also present, with a prevalence of 23.3% and effect sizes between *d* = 0.42–0.53 for VP or low‐birth weight children [12, 15]. In our cohort, the regression coefficients of VP birth on fine motor skills are more pronounced than on visual perception (*β* = −0.44, *β* = −0.25, respectively).

(II) Previous studies have established a correlation between fine motor skills and VMI, as well as between visual perception and VMI [13, 16, 17]. Our analysis confirms these findings and provides further insight into the relationship between these variables. Specifically, our results indicate that fine motor skills have a greater explanatory power for VMI than visual perception. Moreover, our analysis reveals that fine motor skills and visual perception are positively associated with VMI, suggesting that individuals with well‐developed fine motor skills and visual perception tend to exhibit better VMI.

(III) While visual perception and fine motor skills are components of VMI, VP and term born children could show difficulties either at the level of component skills, at the level of integrating these skills, both or neither level. By examining the mediating role of component skills, these different scenarios can be distinguished and the heterogeneity in developmental outcomes can be better understood. The findings demonstrate that VP birth is associated with impairments in both visual perception and fine motor skills independently, and that these impairments substantially explain the observed difficulties in VMI. However, the remaining direct effect of VP birth on VMI suggests that beyond deficits in component skills, there may be additional challenges specific to the integration process itself. This pattern indicates that VP children face a ‘double burden’: compromised foundational skills combined with additional difficulties in coordinating these skills for complex visual‐motor tasks. From a clinical perspective, this finding has important implications. Interventions should target not only the component skills but also the integration abilities to comprehensively address the developmental needs of VP children. The effect of VP birth on VMI is significantly mediated by fine motor skills and visual perception, with fine motor skills exhibiting a greater explanatory power. The greater effect of fine motor skills on VMI compared to visual perception (II) remains stable when both mediators are considered in the overall model (III). Fine motor skills, for instance, are crucial in activities of daily life, such as eating, dressing and playing. Deficits in these skills can lead to significant participation barriers. VP children are often enrolled in follow‐up programs, and thus the opportunity for early intervention in fine motor skills is given to positively impact on VMI at preschool age. Research conducted on the individual abilities of children across various age groups has indicated the occurrence of deficits in VP children during early and middle childhood. It is reasonable to hypothesize that these deficits do not resolve but persist [14, 15]. However, the lack of longitudinal studies limits our understanding of whether these difficulties resolve or persist into adolescence and adulthood.

To effectively leverage the brain's plasticity and learning potential, interventions should be initiated during sensitive periods; however, the timing and specific interventions for VMI have yet to be identified [23]. Current knowledge on the effectiveness of occupational therapy for handwriting skills in 4–6 year‐olds is based on non‐randomised studies [24]. These studies suggest that occupational therapy could have a positive impact on child development. In contrast, an observational study showed that preterm children did not benefit from early care programs in terms of their VMI at the age of 3–6 years. However, the authors included only 15 preterm children and information on the content of the program is sparce [25].

In a randomised controlled trial, term born children with developmental coordination disorder at the age of 8–12 years have benefited from occupational therapy with regards to motor skills [26]. Further, randomised controlled studies are urgently needed to evaluate the sustainable effect of treatments. Beyond therapeutic interventions, it is beneficial to guide parents with suggestions for creating an environment that is conducive to their child's learning. This can be facilitated by encouraging even young children to engage in multi‐faceted learning experiences. These include activities that enhance fine motor skills such as independent eating with cutlery, utilising a pincer grasp for picking up small objects, participating in thread games and providing motivation for toddlers to dress and undress with minimal assistance.

These results may also show that it is advisable to examine VP children with difficulties in VMI in a more differentiated way during preschool age to enable individual support. In addition to standardised assessments of basic functions, everyday skills should also be evaluated.

The finding that the mediation model accounts for 52% of the total variation in individual children's visual motor performance suggests that the mediators are capturing a significant proportion of the underlying factors influencing this outcome. However, the fact that the mediators measure very similar domains cross‐sectionally may have contributed to the relatively high proportion of explained variance. It is also possible that there are other contributing factors such as children's gross motor skills, attention and executive functions, which could be explored in future research.

The significance of VMI also extends to academic performance. Longitudinal studies suggest that the VMI of children before school entry bears predictive value for their academic performance in elementary school [27, 28]. Similarly, positive associations between VMI and academic success have been discovered in neurotypical second‐grade students [29]. Longitudinal studies are needed to investigate specific developmental cascades explaining VMI throughout school age.

Further, it is essential to recognise the uncertainty and rapid rate of change in today's digital era. We can't reliably predict whether traditional handwriting will give way entirely to keyboarding or digital interactions for all children in the future. Could fine motor difficulties potentially be evident during keyboarding as well? On the other hand, given their broader relevance to everyday life, fine motor skills are a crucial area deserving more in‐depth analysis and intervention, beyond their application in writing.

The present study's strengths lie in the homogeneity of its cohort, comprising low‐risk VP children born after 2010 and assessed in 2017–2018, reflecting relatively contemporary neonatal care and developmental outcomes. While neonatal intensive care continues to advance, recent evidence suggests that the prevalence of mild neurodevelopmental delays in VP children has remained stable despite improvements in survival rates [5]. The developmental relationships we identified between visual perception, fine motor skills and VMI likely reflect fundamental neurodevelopmental processes associated with preterm birth rather than cohort‐specific effects. The initial publication of this cohort contributed data on performance and prevalence of developmental delays in visual motor integration, fine motor skills and visual perception to the international literature [12]. The findings are consistent with reports from diverse geographical settings [14, 15], suggesting good generalizability. This study combines an investigation of visual perception, fine motor and VMI, while controlling for confounders such as parental education and child sex. Previous studies have either not investigated all three skills simultaneously, thus lacking documentation of a complete developmental profile, or they have not included a control group [15, 30]. The standardised regression coefficients and the mediation effects allow for the first time a better understanding of the relationship between VP birth and VMI, fine motor skills and visual perception. Nevertheless, replication studies in contemporary cohorts from diverse populations would further strengthen the evidence base for these clinical recommendations.

However, the study has limitations. The cross‐sectional nature of our assessment limits causal inference about the developmental sequence of these skills. Longitudinal studies measuring visual perception, fine motor skills and VMI at multiple time points would provide stronger evidence for the developmental pathways we propose and could clarify whether deficits in component skills precede or co‐develop with integration difficulties. While very preterm children with severe cognitive impairments were excluded, we did not include IQ as a mediator in our model. General cognitive ability correlates strongly with visual motor integration in extremely preterm children [31] highlighting the multifaceted nature of neurodevelopmental outcomes in this population. Our focus on visual perception and fine motor skills was intentional, as these represent specific, modifiable domains commonly targeted in clinical interventions. Nevertheless, future research should examine the interrelationships between general cognition, component skills and visual motor integration to provide a more comprehensive understanding of developmental pathways in very preterm children. Families who participated may have a higher socioeconomic status than those residing in the study catchment area, as evidenced by the absence of parents with low educational levels. Despite these limitations, the study's findings remain noteworthy. Parental education levels did not differ significantly between groups, suggesting minimal bias in the study's results. Finally, we were unable to measure parental support in VMI, fine motor skills and visual perception. This is a crucial area for future research.

## 4 Conclusion

This cohort study shows an association between fine motor skills, visual perception and VMI, and the impact of VP birth on these abilities. Furthermore, visual perception and fine motor skills mediate effects of very preterm birth on children's VMI. These results extend the understanding of VMI and the role of VP birth, whereby studies on replication and longitudinal development are required.

## Author Contributions

**Anne‐Kathrin Dathe:** conceptualization, methodology, formal analysis, investigation, data curation, writing – original draft, writing – review and editing, visualization, project administration. **Julia Jaekel:** conceptualization, methodology, validation, formal analysis, investigation, resources, data curation, writing – original draft, writing – review and editing, visualization, supervision. **Thomas Hoehn:** investigation, resources, writing – review and editing. **Ursula Felderhoff‐Mueser:** conceptualization, resources, writing – review and editing, supervision, funding acquisition. **Britta Maria Huening:** conceptualization, methodology, formal analysis, investigation, data curation, resources, writing – original draft, writing – review and editing, visualization, supervision, funding acquisition.

## Funding

BH received a speaker honorarium by the Chiesi Foundation, which had no influence of any aspect of the study. Open access publication was enabled by the Open Access Publication Fund of the University of Duisburg‐Essen.

## Conflicts of Interest

The authors declare no conflicts of interest.

## Data Availability

The data that support the findings of this study are available from the corresponding author upon reasonable request.
